# Worth it or not? Primary tumor resection for stage IV pancreatic cancer patients: A SEER‐based analysis of 15,836 cases

**DOI:** 10.1002/cam4.4147

**Published:** 2021-07-21

**Authors:** Ningzhen Fu, Yu Jiang, Yuanchi Weng, Hao Chen, Xiaxing Deng, Baiyong Shen

**Affiliations:** ^1^ Department of General Surgery Pancreatic Disease Center Ruijin Hospital affiliated to Shanghai Jiao Tong University School of Medicine Shanghai China; ^2^ Shanghai Jiao Tong University School of Medicine Research Institute of Pancreatic Disease Shanghai China; ^3^ State Key Laboratory of Oncogenes and Related Genes Shanghai China; ^4^ Shanghai Jiao Tong University Institute of Translational Medicine Shanghai China

**Keywords:** cancer‐specific survival (CSS), metastatic disease, overall survival (OS), pancreatic cancer (PC), primary tumor resection (PTR)

## Abstract

**Background:**

Primary tumor resection (PTR) as a treatment option for patients with stage IV pancreatic cancer (PC) is controversial.

**Patients and methods:**

Stage IV PC patients, with treatment data from the National Cancer Institute's Surveillance, Epidemiology, and End Results (SEER), were screened. The main outcomes were overall survival (OS) and cancer‐specific survival (CSS).

**Results:**

We enrolled 15,836 stage IV PC patients in this study. Propensity score‐matched analyses revealed improved OS and CSS of patients receiving chemotherapy plus PTR versus chemotherapy (median survival time [MST_OS_]: 13 vs. 9 months, *p* = 0.024; MST_CSS_: 14 vs. 10 months, *p* = 0.035), and chemoradiotherapy plus PTR versus chemoradiotherapy (MST_OS_: 14 vs. 7 months, *p* = 0.044; MST_CSS_: 14 vs. 7 months, *p* = 0.066). Multivariate adjusted analyses further confirmed these results. Stratified with different metastatic modalities, multivariate analyses suggested that PTR significantly improved the OS and CSS among patients with ≤1 metastatic organ, and that patients with brain metastasis might not benefit from chemotherapy treatment.

**Conclusion:**

PTR improves the OS and CSS of stage IV PC patients on the basis of chemotherapy or chemoradiotherapy, provided that the metastases involve ≤1 organ. Chemotherapy, however, should be carefully considered in patients with metastases involving the brain.

## Graphical abstract

Primary site resection should be recommended for stage IV pancreatic cancer patients, provided that synchronous chemotherapy/chemoradiotherapy was administrated. Chemotherapy, however, may not bring survival benefits for those with brain metastases.

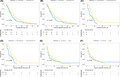

## 1 BACKGROUND

Pancreatic cancer (PC) is well‐known for its high mortality rate and poor prognosis, causing 466,003 deaths in 2020.^1^ Radical surgery is the only treatment option for this malignant disease. In 2020, 495,773 patients were diagnosed with PC,^1^ and it was reported that approximately 60% of newly diagnosed PCs were metastatic.^2^ Even with the current advancements in chemotherapy and radiotherapy, these patients can hardly survive for more than 1 year.^3^, ^4^, ^5^, ^6^, ^7^ For these patients, neither the National Comprehensive Cancer Network (NCCN)^8^ nor the American Society of Clinical Oncology (ASCO) recommend resection of the primary tumor.^9^ Nevertheless, radical surgery to treat primary or metastatic sites has been accepted and conducted in an increasing number of metastatic tumors, including but not limited to neuroendocrine neoplasm, breast cancer, and colorectal cancer.^10^, ^11^, ^12^, ^13^ Thus far, primary tumor resection (PTR) for stage IV PC remains controversial.^14^, ^15^, ^16^, ^17^, ^18^, ^19^, ^20^, ^21^, ^22^ A previous study by our group discovered that surgery helped to prolong the overall survival (OS) of patients with stage IV PC.^14^ Some also advocated PTR and emphasized the importance of patient selection,^15^, ^20^ while others reported no survival benefits from PTR.^19^, ^22^ Due to the violation of established guidelines, approval for clinical trials to study this issue is difficult to acquire. Therefore, there is no strong evidence to clarify the problem. Thus, we turn to the Surveillance, Epidemiology, and End Results (SEER) database, to evaluate the value of PTR among stage IV PC patients.

The SEER is a clinical database that collects cancer incidence, prevalence, and survival data from US cancer registries that cover approximately 34.6% of the US population.^23^ With the large‐volume multi‐center database, we screened eligible stage IV PC patients with definite metastasis, follow‐up, and treatment data. OS and cancer‐specific survival (CSS) were utilized as the main outcomes, and analyzed with multiple statistical methods to determine the value of PTR in the treatment of stage IV PC patients.

## 2 PATIENTS AND METHODS

### 2.1 Patients

The eligibility criteria for patients in this study were patients older than 18 years, who had stage IV PC with metastatic disease at diagnosis, from 1975 to 2016. All patients were pathologically diagnosed with primary malignant tumors of the pancreas using ICD‐O‐3 codes of 8140/3 or 8500/3. The primary site‐labeled column was C25.0–C25.3, and C25.7–C25.9. In total, we identified 98.949 patients with PC, who had recorded treatment information. A total of 19,400 patients with stage IV PC were screened out with clear metastatic site information. Excluding those with unknown race, unknown primary site surgery, unknown regional/distant site surgery data, and no survival months, 15,836 patients were enrolled in our study. The final cohort was then divided into different comparison patterns for the purpose of synchronous presentations, including those who received chemotherapy with or without PTR (*N* = 9515), those undergoing chemoradiation with or without PTR (*N* = 699), and those who received no treatment versus PTR only (*N* = 5403). (Figure S1) It was noteworthy that we lacked detailed information, such as timing of treatments, whether the therapy paradigms were sequential or synchronized was unclear.

### 2.2 Methods

OS and CSS were calculated using the Kaplan–Meier method, univariate comparisons relied on the log‐rank test, and unadjusted Cox models when necessary. Cox proportional hazard regression models adjusted for other variables were applied to calculate adjusted hazard ratios, 95% confidence intervals, and *p* values.

Propensity score matching (PSM) methods were used to adjust differences among the aforementioned comparison patterns in our study. The caliper was set to 0.05, with a matching ratio of 1:1. The matched covariates are presented in the corresponding tables.

As timing of the enrollment relative to surgical resection differed, sequential landmark analyses were implemented to evaluate survival in varied settings for patients surviving a minimum of 0.5, 1, 2, and 3 years from diagnosis, to eliminate “time‐to‐treatment” bias.

A forest plot was generated to evaluate the effect of PTR‐combined therapy on OS by subgroups including age, race, sex, grade, T/N stage, tumor location, metastatic site, and number of involved organs, and further, to explore the potential candidates suitable for PTR.

Statistical analyses were performed using SPSS version 26, and R version 3.6.3. Normally distributed continuous variables are displayed as average (standard deviation, SD), while non‐normally distributed continuous variables are presented as median (Q1–Q3). The Kolmogorov–Smirnov test was used for normality testing of continuous variables. The Wilcoxon (Mann–Whitney) and Kruskal–Wallis tests were used to evaluate continuous variables. Categorical variables are presented as percentages and analyzed using Pearson's test. A two‐sided *p* value of less than 0.05 was considered to be statistically significant.

## 3 RESULTS

Among the 15,836 stage IV PC patients, the median follow‐up was 33 months (median survival time [MST_OS_], 4 months; MST_CSS_, 5 months). According to the different treatment types, patients were categorized into three groups: chemotherapy with or without PTR (*N* = 9,515), chemoradiation with or without PTR (*N* = 699), and no treatment versus PTR only (*N* = 5,403). All patient characteristics are presented in Table 1.

**TABLE 1:** The baseline and demographic characteristics of total cohort and different comparison patterns

	All	Comparison pattern 1			Comparison pattern 2			Comparison pattern 3		
	(*N* = 15,836)	Chemotherapy (*N* = 9190)	Chemotherapy plus PTR (*N* = 325)	*p*	Chemoradiotherapy (*N* = 636)	Chemoradiotherapy plus PTR (*N* = 63)	*p*	No treatment (*N* = 5,231)	PTR only (*N* = 172)	*p*
Age	67 (59–75)	66 (58–73)	65 (58–71)	0.107	64 (57–72)	62 (57–68)	0.12	71 (62–79)	70 (62–78)	0.174
Female	7419 (46.8)	4211 (45.8)	142 (43.7)	0.449	284 (44.7)	34 (54)	0.157	2552 (48.8)	97 (56.4)	0.05
Race										
White	12,549 (79.2)	7432 (80.9)	259 (79.7)	0.064	492 (77.4)	54 (85.7)	0.06	3998 (76.4)	143 (83.1)	0.121
Black	2097 (13.2)	1140 (12.4)	34 (10.5)		86 (13.5)	2 (3.2)		786 (15)	19 (11)	
Other	1190 (7.5)	618 (6.7)	32 (9.8)		58 (9.1)	7 (11.1)		447 (8.5)	10 (5.8)	
Grade										
I	280 (1.8)	131 (1.4)	17 (5.2)	<0.001	17 (2.7)	3 (4.8)	<0.001	94 (1.8)	16 (9.3)	<0.001
II	1612 (10.2)	870 (9.5)	124 (38.2)		66 (10.4)	27 (42.9)		439 (8.4)	72 (41.9)	
III	1842 (11.6)	972 (10.6)	129 (39.7)		77 (12.1)	21 (33.3)		558 (10.7)	59 (34.3)	
IV	65 (0.4)	34 (0.4)	1 (0.3)		6 (0.9)	0 (0)		19 (0.4)	3 (1.7)	
Unknown	12,037 (76)	7183 (78.2)	54 (16.6)		470 (73.9)	12 (19)		4121 (78.8)	22 (12.8)	
T stage										
1	750 (4.7)	424 (4.6)	20 (6.2)	<0.001	23 (3.6)	5 (7.9)	0.001	251 (4.8)	14 (8.1)	<0.001
2	4951 (31.3)	2895 (31.5)	151 (46.5)		182 (28.6)	27 (42.9)		1575 (30.1)	61 (35.5)	
3	4701 (29.7)	2868 (31.2)	109 (33.5)		156 (24.5)	22 (34.9)		1437 (27.5)	62 (36)	
4	2423 (15.3)	1468 (16.0)	31 (9.5)		146 (23)	7 (11.1)		712 (13.6)	20 (11.2)	
Unknown	3011 (19)	1535 (16.7)	14 (4.3)		129 (20.3)	2 (3.2)		1256 (24)	15 (8.7)	
N stage										
0	322 (2)	124 (1.3)	66 (20.3)	<0.001	17 (2.7)	16 (25.4)	<0.001	63 (1.2)	34 (19.8)	<0.001
1	332 (2.1)	95 (1.0)	103 (31.7)		9 (1.4)	19 (30.2)		52 (1)	53 (30.8)	
2	168 (1.1)	3 (0.0)	93 (28.6)		0 (0)	17 (27)		5 (0.1)	49 (28.5)	
Unknown	15014 (94.8)	8968 (97.6)	63 (19.4)		610 (95.9)	11 (17.5)		5111 (97.7)	36 (20.9)	
Tumor location										
Head	5574 (35.2)	3109 (33.8)	183 (56.3)	<0.001	248 (39)	38 (60.3)	0.001	1829 (35)	94 (54.7)	<0.001
Bodytail	5913 (37.3)	3.656 (39.8)	110 (33.8)		220 (34.6)	19 (30.2)		1787 (34.2)	54 (31.4)	
Overlapping	1470 (9.3)	910 (9.9)	17 (5.2)		52 (8.2)	5 (7.9)		465 (8.9)	8 (4.7)	
Other	2879 (18.2)	1515 (16.5)	15 (4.6)		116 (18.2)	1 (1.6)		1150 (22)	16 (9.3)	
Msite bone	1133 (7.2)	509 (5.5)	6 (1.8)	0.004	209 (32.9)	3 (4.8)	<0.001	293 (5.6)	6 (3.5)	0.233
Msite brain	102 (0.6)	29 (0.3)	0 (0)	0.311	25 (3.9)	0 (0)	0.109	28 (0.5)	0 (0)	0.336
Msite liver	12,151 (76.7)	7265 (79.1)	185 (56.9)	<0.001	375 (59)	24 (38.1)	0.001	4072 (77.8)	90 (52.3)	<0.001
Msite lung	3107 (19.6)	1816 (19.8)	31 (9.5)	<0.001	158 (24.8)	5 (7.9)	0.002	1021 (19.5)	20 (11.6)	0.01
Msite number										
0	2345 (14.8)	1226 (13.3)	114 (35.1)	<0.001	125 (19.7)	32 (50.8)	<0.001	762 (14..6)	65 (37.8)	<0.001
1	10,840 (68.5)	6473 (70.4)	200 (61.5)		312 (49.1)	30 (47.6)		3620 (69.2)	98 (57)	
2	2314 (14.6)	1332 (14.5)	11 (3.4)		144 (22.6)	1 (1.6)		755 (14.4)	9 (5.2)	
3	323 (2)	154 (1.7)	0 (0)		53 (8.3)	0 (0)		92 (1.8)	0 (0)	
4	14 (0.1)	5 (0.1)	0 (0)		2 (0.3)	0 (0)		2 (0)	0 (0)	
Insurance status										
Insured	15,201 (96)	8874 (96.6)	316 (97.2)	0.806	610 (95.9)	60 (95.2)	0.967	4971 (95)	165 (95.9)	0.79
Uninsured	421 (2.7)	215 (2.3)	6 (1.8)		17 (2.7)	2 (3.2)		163 (3.1)	5 (2.9)	
Unknown	214 (1.4)	101 (1.1)	3 (0.9)		1.4 (9)	1 (1.6)		97 (1.9)	2 (1.2)	
Married	9056 (57.2)	5725 (62.3)	226 (69.5)	0.008	373 (58.6)	38 (60.3)	0.797	2.472 (47.3)	105 (61)	<0.001
Chemotherapy	10,214 (64.5)	\	\		\	\		\	\	
Radiotherapy	918 (5.8)	\	\		\	\		\	\	
PTR	562 (3.5)	\	\		\	\		\	\	
Distant/reginal site resection	963 (6.1)	421 (4.6)	110 (33.8)	<0.001	54 (8.5)	24 (38.1)	<0.001	272 (5.2)	66 (38.4)	<0.001

Abbreviations: Msite, metastatic site; PTR, primary tumor resection.

**FIGURE 1:**
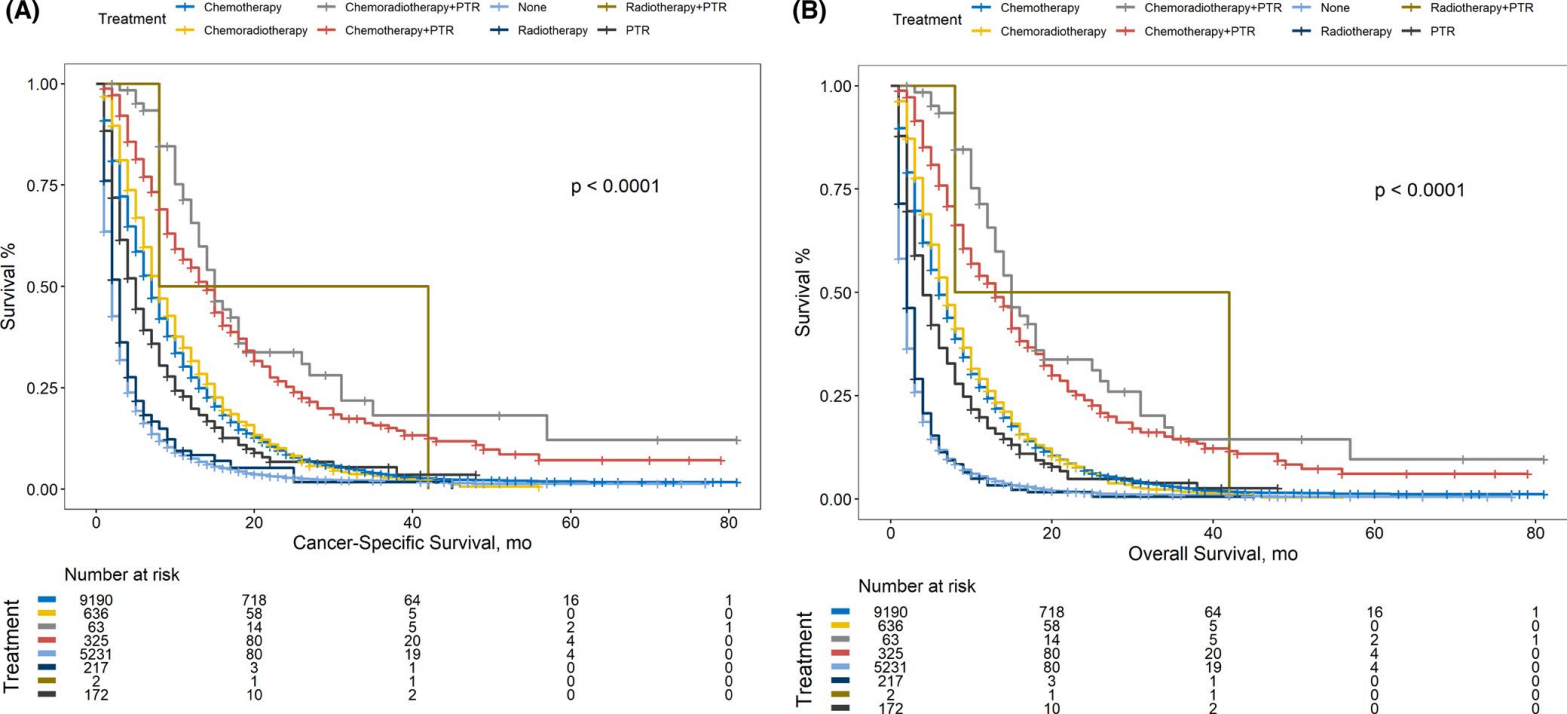
(A) Comparison of OS among stage IV PC patients receiving different treatment modalities. (B) Comparison of CSS among stage IV PC patients receiving different treatment modalities

Kaplan–Meier analysis was performed targeting OS and CSS categorized with different therapy modalities. (Figure 1) MST was calculated and listed in order from high to low: OS: chemoradiotherapy plus PTR, 15 months; chemotherapy plus PTR, 13 months; radiotherapy plus PTR, 8 months; chemoradiotherapy, 7 months; chemotherapy, 6 months; PTR, 4 months; radiotherapy, 2 months; no treatment, 2 months; CSS: chemoradiotherapy plus PTR, 15 months; chemotherapy plus PTR, 14 months; chemoradiotherapy, 8 months; radiotherapy plus PTR, 8 months; chemotherapy, 7 months; PTR, 5 months; radiotherapy, 3 months; no treatment, 2 months. The PTR intervention significantly improved OS among all three comparison patterns. (Figure 1) Similarly, beneficial outcomes of PTR were also observed when analyzing CSS. (Figure 1).

**FIGURE 2:**
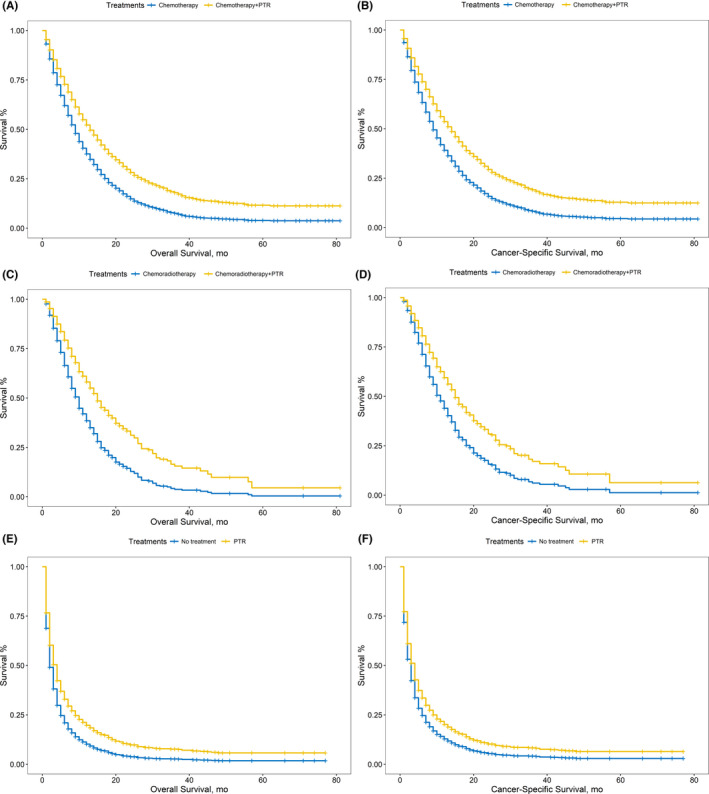
(A) Comparison of OS between patients receiving chemotherapy and chemotherapy plus PTR with covariates adjusted. (B) Comparison of CSS between patients receiving chemotherapy and chemotherapy plus PTR with covariates adjusted. (C) Comparison of OS between patients receiving chemoradiotherapy and chemoradiotherapy plus PTR with covariates adjusted. (D) Comparison of CSS between patients receiving chemoradiotherapy and chemoradiotherapy plus PTR with covariates adjusted. (E) Comparison of OS between patients receiving with covariates adjusted. (A) Comparison of CSS between patients receiving no treatments and PTR only with covariates adjusted

**FIGURE 3:**
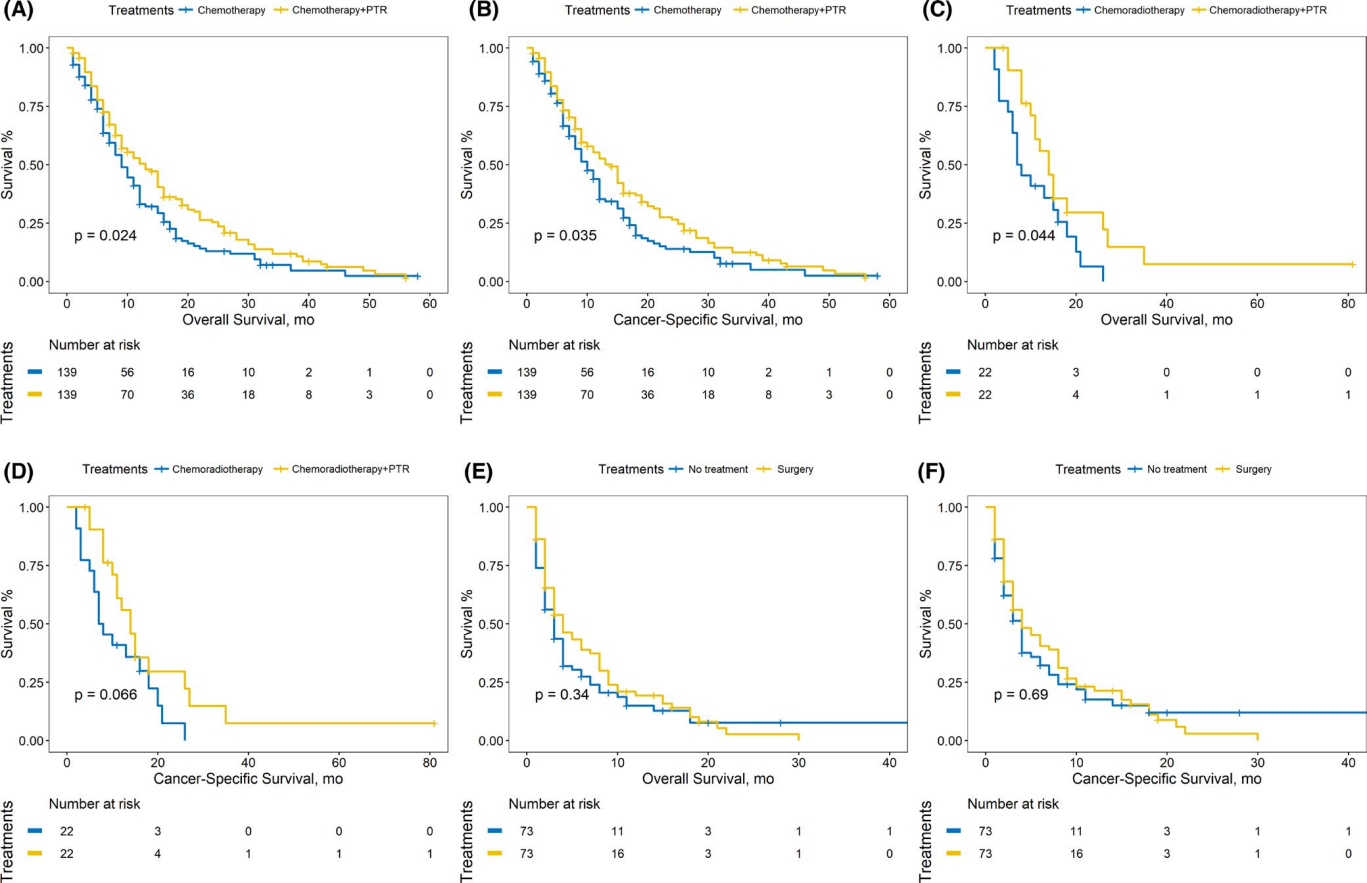
(A) Comparison of OS between patients receiving chemotherapy and chemotherapy plus PTR after PSM. (B) Comparison of CSS between patients receiving chemotherapy and chemotherapy plus PTR after PSM. (C) Comparison of OS between patients receiving chemoradiotherapy and chemoradiotherapy plus PTR after PSM. (D) Comparison of CSS between patients receiving chemoradiotherapy and chemoradiotherapy plus PTR after PSM. (E) Comparison of OS between patients receiving after PSM (A) Comparison of CSS between patients receiving no treatments and PTR only after PSM

After adjusting for confounding covariates, PTR also significantly improved the OS and CSS in different comparison patterns. (Figure 2, Table 2) PSM was performed to reduce the confounding effects of covariates for three pairs of comparison patterns in addition. The matched cohorts are shown in Table 3. Compared with PTR‐absent treatments, additional PTR significantly improved the OS and CSS of patients with stage IV PC. (Figure 3; chemotherapy plus PTR vs. chemotherapy: OS: 13 vs. 9 months, *p* = 0.024; CSS: 14 vs. 10 months, *p* = 0.035; chemoradiotherapy plus PTR vs. chemoradiotherapy: OS: 14 vs. 7 months, *p* = 0.044; CSS: 14 vs. 7 months, *p* = 0.066; PTR only vs. no treatment: OS: 4 vs. 3 months, *p* = 0.34; CSS: 4 vs. 4 months, *p* = 0.69).

**TABLE 2:** Univariate and multivariate survival analyses of different variables aiming at OS and CSS for patients with stage IV PC

	Univariate				Multivariate			
	OS		CSS		OS		CSS	
	HR (95% CI)	*p*	HR (95% CI)	*p*	aHR (95% CI)	*p*	aHR (95% CI)	*p*
Age	1.016 (1.014–1.017)	<0.001	1.015 (1.013–1.017)	<0.001	1.010 (1.008–1.012)	<0.001	1.009 (1.008–1.011)	<0.001
Female	0.987 (0.971–1.004)	0.125	0.992 (0.975–1.010)	0.374				
Race		0.008		0.226		0.084		
White	Ref		Ref		Ref			
Black	1.079 (1.027–1.133)	0.002	1.021 (0.982–1.062)	0.297	1.058 (1.007–1.112)	0.027		
Other	1.032 (0.969–1.099)	0.328	1.002 (0.957–1.049)	0.936	1.015 (0.952–1.081)	0.655		
Grade		<0.001		<0.001		<0.001		<0.001
I	Ref		Ref		Ref		Ref	
II	1.130 (0.986–1.294)	0.079	1.119 (0.972–1.290)	0.119	1.283 (1.119–1.471)	<0.001	1.256 (1.089–1.448)	0.002
III	1.420 (1.242–1.625)	<0.001	1.403 (1.219–1.614)	<0.001	1.612 (1.408–1.846)	<0.001	1.565 (1.359–1.802)	<0.001
IV	1.287 (0.959–1.729)	0.093	1.219 (0.890–1.669)	0.218	1.282 (0.954–1.722)	0.100	1.191 (0.869–1.632)	0.276
Unknown	1.430 (1.259–1.624)	<0.001	1.393 (1.220–1.590)	<0.001	1.447 (1.273–1.645)	<0.001	1.402 (1.227–1.602)	<0.001
T stage		<0.001		<0.001		<0.001		<0.001
1	Ref		Ref		Ref		Ref	
2	1.112 (1.023–1.209)	0.013	1.119 (1.025–1.221)	0.012	1.139 (1.047–1.238)	0.002	1.139 (1.044–1.244)	0.003
3	1.226 (1.127–1.333)	<0.001	1.243 (1.138–1.356)	<0.001	1.243 (1.142–1.353)	<0.001	1.248 (1.142–1.363)	<0.001
4	1.145 (1.048–1.250)	0.003	1.157 (1.055–1.268)	0.002	1.196 (1.095–1.307)	<0.001	1.206 (1.099–1.323)	<0.001
Unknown	1.422 (1.304–1.550)	<0.001	1.254 (1.144–1.374)	<0.001	1.296 (1.185–1.417)	<0.001	1.182 (1.075–1.299)	0.001
N stage		<0.001		<0.001		<0.001		<0.001
0	Ref		Ref	<0.001	Ref		Ref	
1	1.081 (0.910–1.286)	0.375	1.112 (0.928–1.333)	0.248	1.159 (0.974–1.379)	0.096	1.199 (0.999–1.438)	0.051
2	1.120 (0.911–1.378)	0.282	1.144 (0.921–1.420)	0.224	1.427 (1.143–1.781)	0.002	1.403 (1.114–1.768)	0.004
Unknown	1.881 (1.659–2.134)	<0.001	1.878 (1.645–2.144)	<0.001	1.435 (1.251–1.645)	<0.001	1.469 (1.271–1.697)	<0.001
Tumor location		<0.001		<0.001		<0.001		<0.001
Head	0.908 (0.873–0.944)	<0.001	0.897 (0.861–0.934)	<0.001	0.914 (0.878–0.952)	<0.001	0.91 (0.873–0.949)	<0.001
Bodytail	Ref		Ref		Ref		Ref	
Overlapping	1.030 (0.970–1.094)	0.339	1.047 (0.983–1.114)	0.152	0.993 (0.943–1.046)	0.792	1.016 (0.955–1.082)	0.611
Other	1.154 (1.101–1.209)	<0.001	1.017 (0.966–1.070)	0.521	1.000 (0.941–1.062)	0.996	0.922 (0.872–0.976)	0.005
Msite bone	1.251 (1.175–1.333)	<0.001	1.168 (1.091–1.251)	<0.001	1.157 (0.992–1.35)	0.064	1.132 (0.952–1.346)	0.159
Msite brain	1.469 (1.201–1.795)	<0.001	1.264 (1.006–1.589)	0.045	1.309 (1.057–1.62)	0.014	1.228 (0.964–1.565)	0.096
Msite liver	1.241 (1.291–1.343)	<0.001	1.323 (1.269–1.380)	<0.001	1.313 (1.132–1.523)	<0.001	1.351 (1.143–1.596)	<0.001
Msite lung	1.114 (1.069–1.162)	<0.001	1.095 (1.048–1.145)	<0.001	1.007 (0.868–1.168)	0.923	1.009 (0.854–1.192)	0.915
Msite number		<0.001		<0.001		<0.001		<0.001
0	Ref		Ref		Ref		Ref	
1	1.234 (1.177–1.295)	<0.001	1.249 (1.187–1.314)	<0.001	0.947 (0.814–1.102)	0.480	0.928 (0.783–1.1)	0.388
2	1.537 (1.446–1.634)	<0.001	1.551 (1.454–1.655)	<0.001	1.104 (0.827–1.472)	0.502	1.091 (0.788–1.509)	0.601
3	1.977 (1.754–2.228)	<0.001	1.768 (1.547–2.020)	<0.001	1.309 (0.848–2.021)	0.224	1.178 (0.721–1.924)	0.514
4	2.244 (1.326–3.796)	<0.001	1.993 (1.102–3.606)	0.023				
Insurance status		0.012		0.068		0.004		0.019
Insured	Ref		Ref		Ref		Ref	
Uninsured	1.154 (1.043–1.277)	0.006	1.131 (1.015–1.260)	0.026	1.188 (1.072–1.317)	0.001	1.17 (1.048–1.305)	0.005
Unknown	1.088 (0.942–1.257)	0.253	1.056 (0.904–1.232)	0.493	1.040 (0.900–1.202)	0.596	1.028 (0.881–1.201)	0.723
Married	0.827 (0.800–0.855)	<0.001	0.846 (0.817–0.876)	<0.001	0.915 (0.884–0.947)	<0.001	0.922 (0.89–0.956)	<0.001
Chemotherapy	0.405 (0.391–0.420)	<0.001	0.421 (0.406–0.437)	<0.001	0.418 (0.403–0.434)	<0.001	0.428 (0.412–0.445)	<0.001
Radiotherapy	0.828 (0.771–0.888)	<0.001	0.790 (0.732–0.852)	<0.001	0.915 (0.849–0.985)	0.018	0.888 (0.82–0.961)	0.003
PTR	0.520 (0.473–0.572)	<0.001	0.543 (0.493–0.599)	<0.001	0.704 (0.613–0.807)	<0.001	0.732 (0.635–0.845)	<0.001
Distant/reginal site resection	0.762 (0.710–0.818)	<0.001	0.740 (0.687–0.798)	<0.001	0.900 (0.836–0.968)	0.005	0.876 (0.81–0.947)	0.001

Abbreviations: aHR, adjusted hazard ratio; CI, confidence interval; CSS, cancer‐specific survival; HR, hazard ratio; Msite, metastatic site; OS, overall survival; *p*, *p* value; PTR, primary tumor resection.

**TABLE 3:** Baseline and demographic characteristics of different comparison patterns after PSM

	Comparison pattern 1			Comparison pattern 2			Comparison pattern 3		
	Chemotherapy (*N* = 139)	Chemotherapy plus PTR (*N* = 139)	*p*	Chemoradiotherapy (*N* = 22)	Chemoradiotherapy plus PTR (*N* = 22)	*p*	No treatment (*N* = 73)	PTR only (*N* = 73)	*p*
Age	64 (57–71)	65 (58–71)	0.6	63 (54–72)	61 (56–67)	0.481			
Female	72 (51.8)	59 (42.4)	0.149	13 (59.1)	10 (45.5)	0.546	41 (56.2)	42 (57.5)	0.867
Race									
White	107 (77.0)	108 (77.7)	0.805	20 (90.9)	20 (90.9)	1	56 (76.7)	56 (76.7)	0.585
Black	16 (11.5)	18 (12.9)		1 (4.5)	1 (4.5)		8 (11)	11 (15.1)	
Other	16 (11.5)	13 (9.4)		1 (4.5)	1 (4.5)		9 (12.3)	9 (12.3)	
Grade									
I	7 (5.0)	8 (5.8)	0.656	2 (9.1)	1 (4.5)	0.727	6 (8.2)	7 (9.6)	0.222
II	40 (28.8)	43 (30.9)		4 (18.2)	7 (31.8)		22 (30.1)	27 (37)	
III	35 (25.2)	41 (29.5)		5 (22.7)	4 (18.2)		12 (16.4)	18 (24.7)	
IV	1 (0.7)	0 (0.0)		0 (0)	0 (0)		2 (2.7)	0 (0)	
Unknown	56 (40.3)	47 (33.8)		11 (50.0)	10 (45.5)		31 (42.5)	21 (28.8)	
T stage									
1	7 (5.0)	10 (7.2)	0.608	2 (9.1)	1 (4.5)	0.962	7 (9.6)	4 (5.5)	0.825
2	53 (38.1)	61 (43.9)		10 (45.5)	9 (40.9)		27 (37)	24 (32.9)	
3	42 (30.2)	40 (28.8)		4 (18.2)	5 (22.7)		17 (23.3)	19 (26)	
4	25 (18.0)	17 (12.2)		4 (18.2)	5 (22.7)		11 (15.1)	12 (16.4)	
Unknown	12 (8.6)	11 (7.9)		2 (9.1)	2 (9.1)		11 (15.1)	14 (19.2)	
N stage									
0	40 (28.8)	42 (30.2)	0.58	8 (36.4)	10 (45.5)	0.812	17 (23.3)	15 (20.5)	0.926
1	38 (27.3)	29 (20.9)		4 (18.2)	3 (13.6)		20 (27.4)	18 (24.7)	
2	3 (2.2)	5 (3.6)		0 (0.0)	0 (0.0)		4 (5.5)	4 (5.5)	
Unknown	58 (41.7)	63 (45.3)		10 (45.5)	9 (40.9)		32 (43.8)	36 (49.3)	
Tumor location									
Head	78 (56.1)	73 (52.5)	0.834	16 (72.7)	13 (59.1)	0.65	36 (49.3)	35 (47.9)	0.974
Bodytail	42 (30.2)	48 (34.5)		5 (22.7)	7 (31.8)		23 (31.5)	22 (30.1)	
Overlapping	9 (6.5)	7 (5.0)		1 (4.5)	1 (4.5)		3 (4.1)	4 (5.5)	
Other	10 (7.2)	11 (7.9)		0 (0.0)	1 (4.5)		11 (15.1)	12 (16.4)	
Msite bone	1 (0.7)	3 (2.2)	0.615	1 (4.5)	0 (0.0)	1	2 (2.7)	3 (4.1)	0.649
Msite brain	0 (0.0)	0 (0.0)	\	0 (0.0)	0 (0.0)	\	0 (0)	0 (0)	\
Msite liver	76 (54.7)	90 (64.7)	0.112	12 (54.5)	11 (50.0)	1	34 (46.6)	40 (54.8)	0.321
Msite lung	15 (10.8)	13 (9.4)	0.842	2 (9.1)	1 (4.5)	1	10 (13.7)	9 (12.3)	0.806
Msite number									
0	52 (37.4)	41 (29.5)	0.306	7 (31.8)	10 (45.5)	0.353	32 (43.8)	25 (34.2)	0.413
1	82 (59.0)	90 (64.7)		15 (68.2)	12 (54.5)		36 (49.3)	44 (60.3)	
2	5 (3.6)	8 (5.8)		0 (0.0)	0 (0.0)		5 (6.8)	4 (5.5)	
3	0 (0.0)	0 (0.0)		0 (0.0)	0 (0.0)		0 (0.0)	0 (0.0)	
4	0 (0.0)	0 (0.0)		0 (0.0)	0 (0.0)		0 (0.0)	0 (0.0)	
Insurance status									
Insured	135 (97.1)	135 (97.1)	0.766	20 (90.9)	21 (95.5)	1	70 (95.9)	69 (94.5)	0.843
Uninsured	2 (1.4)	3 (2.2)		0 (0)	0 (0)		1 (1.4)	2 (2.7)	
Unknown	2 (1.4)	1 (0.7)		2 (9.1)	1 (4.5)		2 (2.7)	2 (2.7)	
Married	87 (62.6)	92 (66.2)	0.616	15 (68.2)	13 (59.1)	0.754	43 (58.9)	40 (54.8)	0.616
Distant/reginal site resection	47 (33.8)	37 (26.6)	0.24	7 (31.8)	7 (31.8)	1	21 (28.8)	19 (26)	0.711

PTR,.primary tumor resection. Msite, metastatic site.

**FIGURE 4:**
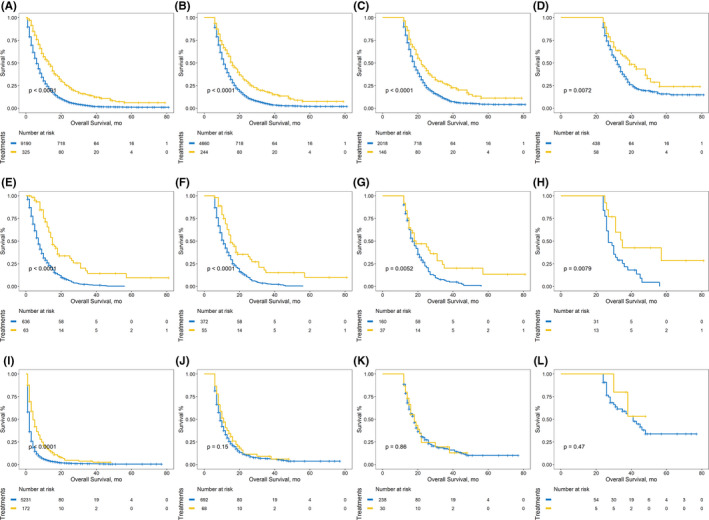
Sequential landmark Kaplan–Meier analyses of OS at ≥0, ≥0.5, ≥1, and ≥2 years between patients receiving chemotherapy and chemotherapy plus PTR, A–D, patients receiving chemoradiotherapy and chemoradiotherapy plus PTR, E–H, patients receiving no treatments and PTR only, I–L. The blue curves referred to the PTR‐absence treatments while the yellow curves referred to the PTR‐combined treatments

To eliminate time‐to‐treatment bias, sequential landmark analyses with landmarks set as 0, 0.5, 1, and 2 years after diagnosis, were performed. (Figure 4, Figure S2) Chemotherapy plus PTR, compared with chemotherapy, improved the OS and CSS in both univariate and multivariate survival analyses. Chemoradiotherapy plus PTR was also advantageous over chemoradiotherapy for CSS and OS. Nevertheless, no significant differences were observed when comparing non‐treatment and PTR‐only patients. (Table S1, Table S2).

**FIGURE 5:**
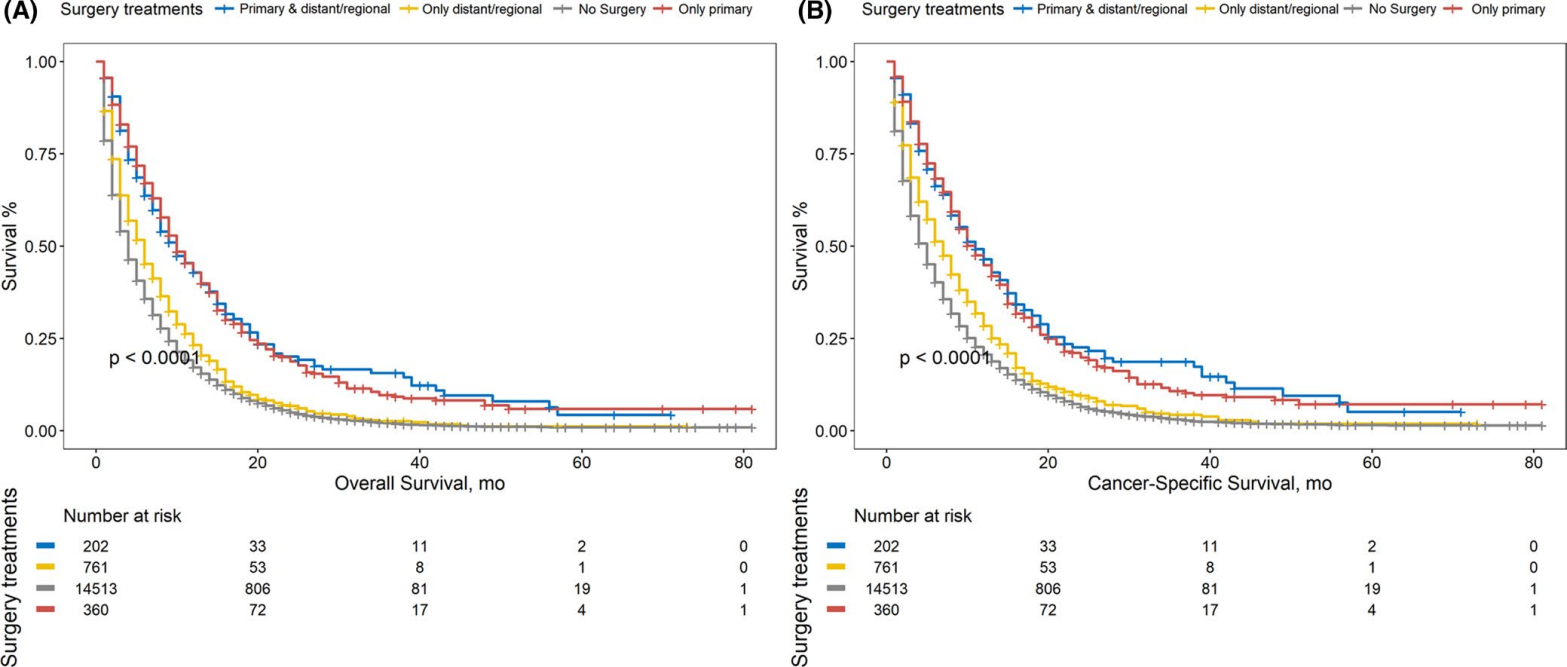
(A) Comparison of OS among stage IV PC patients receiving different surgery treatment modalities. (B) Comparison of CSS among stage IV PC patients receiving different surgery treatment modalities

Except for the primary site, the outcomes of patients receiving distant/regional site resections were also evaluated (Table S3). The MSTs for patients with resections of the primary site and distant/regional site (OS: 10 months; CSS: 11 months), primary site only (OS: 10 months; CSS: 11 months), the distant/regional site only (OS: 6 months; CSS: 7 months), and no surgery (OS: 4 months; CSS: 5 months) were measured, which indicated that surgical interventions increased the OS and CSS in stage IV PC patients. (Figure 5) The pairwise comparisons among the four groups above were all significant (*p* < 0.001) except for that between primary site plus distant/regional site and primary site only (OS: *p* = 0.819, CSS: *p* = 0.570). Additionally, in multivariate analyses, PTR increased the OS and CSS more significantly than distant/regional resection did (Figure 5, multivariate Cox model: primary site vs. no‐surgery: OS, *p* < 0.001; CSS, *p* < 0.001; distant/regional site vs. no‐surgery: OS, *p* = 0.011; CSS, *p* = 0.003). On the basis of primary site resection, distant/regional resection might not result in additional OS/CSS improvements. (Multivariate Cox Model: primary site plus distant/regional site vs. primary site only: OS, *p* = 0.486; CSS, *p* = 0.341).

**FIGURE 6:**
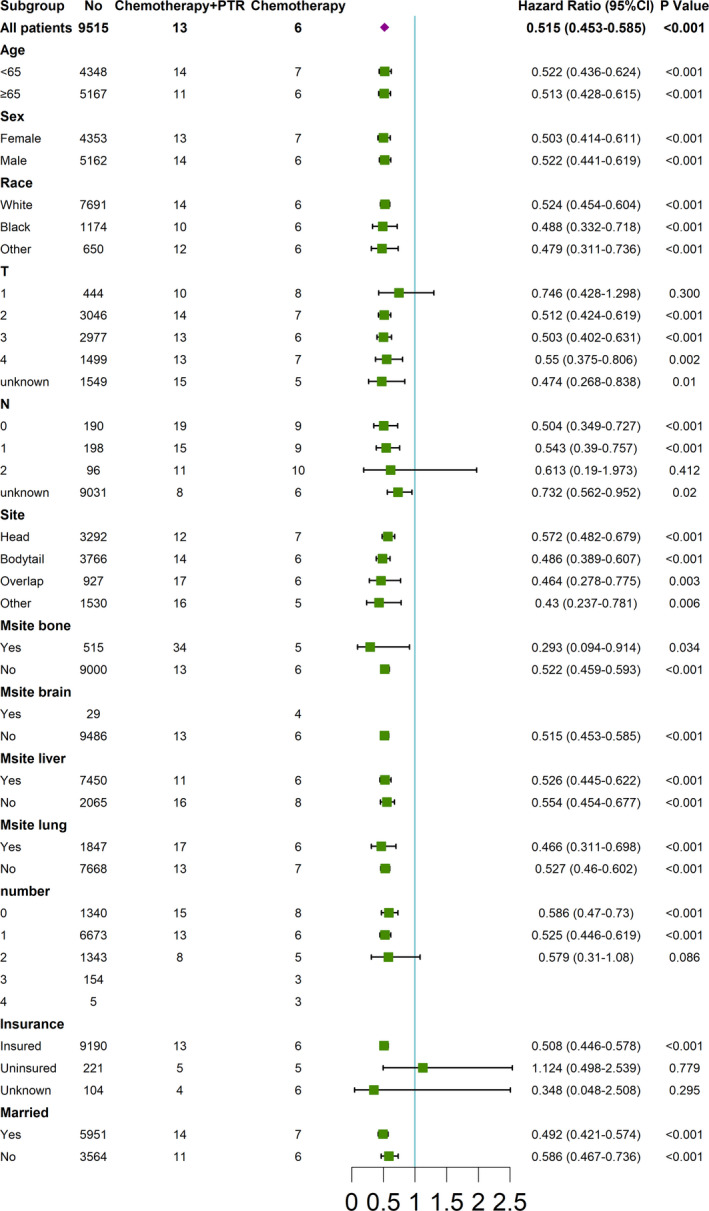
Subgroup analysis of the association between PTR and OS using forest plot (chemotherapy plus PTR vs. chemotherapy). CI, confidence interval; T, tumor stage; N, nodal stage; Msite, metastatic site, including brain, liver, bone and lung; number, metastatic organ summation

A forest plot was constructed to verify whether the OS improvement seen with PTR existed in different subcategories. (Figure 6) It was revealed that insured patients with T2–4, N0–1, and distant metastases involving ≤1 organ, could benefit from PTR regardless of age, sex, race, tumor location, or marital status.

For different organ involvement modalities, we compared the OS and CSS using log‐rank analyses. (Table S4) Stratified with different metastatic modalities, multivariate analyses were performed. (Table 4, Table S5) It was discovered that chemotherapy would no longer ameliorate OS and CSS in patients with brain metastasis. The impact of primary site surgeries was only significant when a single organ was involved. Distant/regional site surgeries were also found to be beneficial in patients with only liver metastasis.

**TABLE 4:** Multivariate Cox regression analyses of different treatments stratified with different metastatic organ modalities considered for overall survival

	Number	PTR			Distant/reginal site resection			Radiotherapy			Chemotherapy		
		aHR	95% CI	*p*	aHR	95% CI	*p*	aHR	95% CI	Sig	aHR	95% CI	*p*
B	229	0.277	0.133–0.579	**0.001**	1.101	0.676–1.794	0.698	1.27	0.954–1.691	0.102	0.339	0.25–0.461	**<0.001**
BC	7				16.102	0.003–79,865.239	0.522	2.007	0.178–22.642	0.573	0.003	0–370.28	0.333
BCH	6				0.998	0.089–11.259	0.999	15.119	0.066–3459.531	0.327	0.005	0–26.38	0.226
BCHP	14				5.146	0.371–71.47	0.222	0.225	0.05–1.008	0.051	0.204	0.043–0.959	0.044
BCP	8				0.551	0.041–7.458	0.654	1.876	0.26–13.526	0.532	0	0–4.237E+163	0.949
BH	444	0.554	0.076–4.048	0.561	0.857	0.433–1.696	0.658	0.909	0.718–1.152	0.43	0.352	0.283–0.439	**<0.001**
BHP	295				0.617	0.197–1.931	0.407	0.928	0.695–1.238	0.61	0.356	0.27–0.47	**<0.001**
BP	130	0.566	0.175–1.832	0.342	1.204	0.67–2.163	0.535	1.108	0.754–1.63	0.601	0.302	0.194–0.47	**<0.001**
C	15				0	0–9.851E13	0.989	0.84	0.238–2.972	0.787	0.336	0.084–1.338	0.122
CH	26				0.655	0.121–3.541	0.623	1.182	0.461–3.032	0.728	0.491	0.201–1.197	0.118
CHP	14				1.45	0.293–7.174	0.649	0.893	0.152–5.257	0.9	0.171	0.019–1.551	0.117
CP	12				2.064	0.156–27.283	0.582	0.186	0.034–1.03	0.054	0.494	0.08–3.045	0.447
H	9657	0.545	0.476–0.624	**<0.001**	0.803	0.722–0.893	**<0.001**	0.869	0.771–0.979	0.021	0.397	0.38–0.416	**<0.001**
HP	1695	0.67	0.4–1.122	0.128	0.941	0.673–1.317	0.724	0.808	0.581–1.126	0.208	0.41	0.368–0.457	**<0.001**
Other	2345	0.586	0.497–0.692	**<0.001**	0.95	0.839–1.077	0.424	0.812	0.686–0.96	0.015	0.384	0.25–0.421	**<0.001**
P	939	0.663	0.458–0.96	0.029	0.943	0.718–1.239	0.674	1.038	0.751–1.436	0.82	0.447	0.386–0.519	**<0.001**

Organ involvement code: P‐lung, C‐brain, H‐liver, and B‐bone. The combination of the letters referred to multi‐organ involvements. Other referred to those IV stage patients with metastatic organs other than four organs mentioned above.

Bold *p* value indicated those less than 0.001.

Abbreviations: aHR, adjusted hazard ratio; CI, confidence interval; *p*, *p* value; PTR, primary tumor resection.

## 4 DISCUSSION

The majority of patients with PC (approximately 60%) were diagnosed with metastatic disease (stage IV).^2^ Current guidelines do not recommend surgery for these patients.^8^, ^9^ And whether these patients should be treated with PTR or not remains a controversy. Some advocate PTR as a treatment modality,^14^, ^15^, ^20^ while others disagreed.^19^, ^22^ Gu et al. compared patients that underwent PTR with those that underwent bypass or exploratory laparotomy only, on the basis of a 3‐year follow‐up.^14^ They reported a significantly prolonged OS rate in patients that underwent PTR. However, the sample size was limited (34 for the PTR group), and adjuvant treatments were not well incorporated into the analysis. The metastatic patterns have not been well explored. The work of Wang et al. was also limited by the lack of data on adjuvant therapy, and metastatic patterns.^15^ Tachezy et al. studied this issue based on 69 cases and meta‐analysis, proving the survival benefits of PTR among hepatic oligometastatic PC patients, especially for patients with tumors located at the head of the pancreas.^20^ Although the neoadjuvant/adjuvant treatment data were listed, they were not well utilized in the analyses. The main limitations of the studies by Dünschede et al, and Gleisner et al. were the sample sizes (23 and 17, respectively), and the nonmention of “time‐to‐treatment” bias.^19^, ^22^ Some were speculative, and proposed that the PTR candidates should be carefully selected, and that the positive impact of surgeries should be further verified.^16^, ^17^, ^18^, ^20^, ^21^ Hitherto, no consensus on this issue has been reached; however, majority still maintain a positive attitude. Previous studies were limited by sample size, lack of adjuvant treatment analyses, insufficient metastatic pattern analyses, and uncorrected “time‐to‐treatment” bias. Thus, based on the SEER database with treatment and metastatic pattern data, we sought to fix the limitations above with multivariate, PSM, stratification, and sequential landmark analyses.

In our study, additional PTR significantly increased the OS and CSS, compared with PTR‐absent treatments in multivariable risk adjustment analyses, based on chemotherapy/chemoradiotherapy. PSM analyses were performed serving as the sensitivity analysis and the complementary verification.

“Time‐to‐treatment” bias arises in comparative research, when survival time is measured from enrollment (e.g., diagnosis), and the receipt of treatment occurs during follow‐up. Patients with poor performance status or significantly aggressive disease, might die too early (before undergoing PTR), which creates an apparent survival disadvantage for patients who do not receive PTR. The time interval between the date of initial diagnosis and the date of surgery, was unknown in the SEER data. The longer the deferral, the greater the “time‐to‐treatment” bias is in favor of PTR. We speculated that “time‐to‐treatment” bias could partially explain the treatment effect estimate in previous studies. In our study, we used the landmark analysis, one of the recommended corrective approaches, to eliminate “time‐to‐treatment” bias, after which PTR with synchronous chemotherapy/chemoradiotherapy was still associated with improved OS and CSS.

As suggested by Drs. Shi, Liu, and Shrikhande, the population for PTR should be carefully selected.^16^, ^17^, ^18^ Forest plot was then performed, which revealed the OS improvement seen with the chemotherapy plus PTR modality compared with chemotherapy only, among different subcategories. It was observed that insured patients with T2–4, N0–1, and distant metastases involving ≤1 organ, could benefit from PTR regardless of age, sex, race, tumor location, or marital status; this could serve as a reference for the screening of PTR candidates. We supposed that there probably existed special oncological behaviors in T1 metastatic patients.

To treat metastatic loci, Dünschede et al. recommended metachronous resection, while Tachezy et al. recommended synchronous resection for liver metastases. Dr. Liu, however, believed that both metachronous and synchronous metastatic resection, improved survival in lung metastases. However, some researchers have recommended nonsurgical treatment options.^24^, ^25^, ^26^ In our study, PTR improved the OS and CSS more than distant/regional site resection. In multivariate Cox analyses, combined distant/regional site resection could not contribute to OS/CSS increase on the basis of PTR.

Due to the insufficiency of studies on metastatic patterns, stratified with different metastatic organ involvement modalities, we verified the impact of different treatments. PTR should be considered only if a single organ is involved, which corresponds to the discoveries in the forest plot analysis. Distant/regional site resection can be applied when only the liver is involved. If the brain is involved, the administration of chemotherapy should be carefully considered. Radiotherapy, however, was not beneficial for patients with stage IV PC; it could be considered as a last complementary choice. The survival prognoses of different metastatic organ modalities varied slightly (MST range ≤8 months). Therefore, the subgroup of metastatic behavior was of no use. In summary, patients with multiorgan or liver involvement were observed to have worse prognoses compared to those with brain, bone, or lung involvement.

This study had some limitations. First, its retrospective nature limited the evidence value of our work. However, our discoveries provide more grounds for ethical committees to approve clinical trials on the subject matter. Second, due to the limitations of the database, details about the treatments, such as the treatment time, chemotherapy regimen, surgery details, and so on were not designed and recorded. These findings should be taken into consideration in future prospective studies. Third, information on the total tumor burden (e.g., size and number of metastatic loci for different involved organs), as well as the response to treatment was lacking. Fourth, selection bias existed because of preference for surgery, for individuals whose general condition was more “acceptable.” Good performance status and personal habits may influence the OS, even though the NCCN guidelines bifurcated the strategies for stage IV PC patients based on performance status, while SEER lacked this information.

Ultimately, “time‐to‐treatment” bias, the metastatic pattern analyses, and combined treatment analyses, which most previous studies neglected, were taken into consideration with a satisfactory sample size. In conclusion, we demonstrated improvements in OS and CSS in patients with stage IV PC. If only one organ is involved, surgery should be performed on the basis of chemotherapy or chemoradiotherapy. The use of chemotherapy as a treatment option in patients with brain metastasis, should be carefully considered due to the lack of significant improvement in OS and CSS in our studies.

## CONFLICTS OF INTEREST

The authors report no conflict of interest.

## ETHICS STATEMENT

This study was exempt from institutional review board approval due to the nature of the study. Because all data were deidentified, patient consent was waived.

## Supporting information

Fig S1

Fig S2

Table S1

Table S2

Table S3

Table S4

Table S5

## Data Availability

The data of our work are available and publicly accessible. The original data comes from the Surveillance, Epidemiology, and End Results (SEER) database.
