# Supplementary figures and images for: Worth it or not? Primary tumor resection for stage IV pancreatic cancer patients: A SEER‐based analysis of 15,836 cases
Source: Cancer Med. 2021 Jul 21;10(17):5948–63. doi: 10.1002/cam4.4147 (PMC8419755; doi:10.1002/cam4.4147)

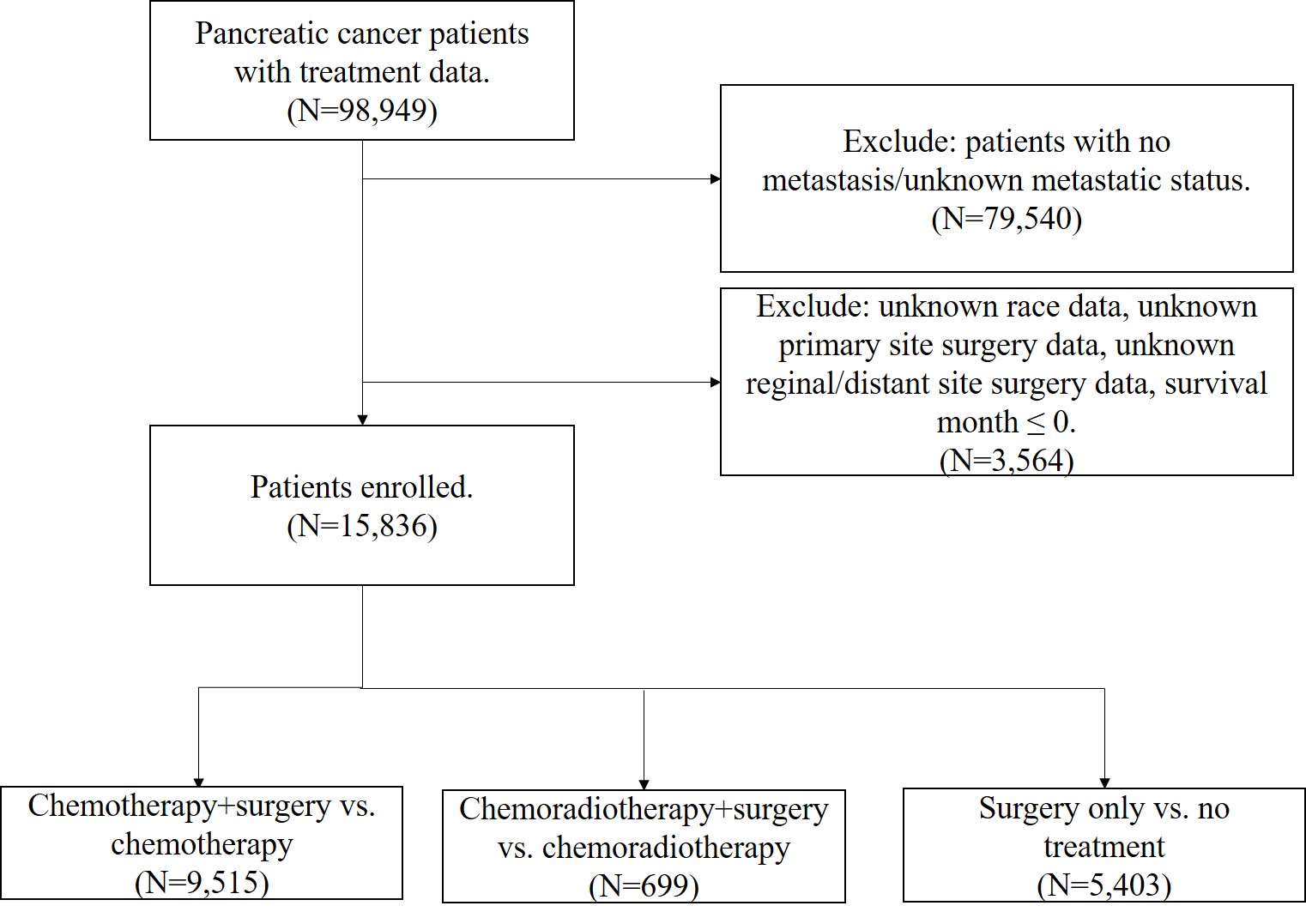

Supplement: Supplementary file 1 — Fig S1 [file CAM4-10-5948-s001.jpg]

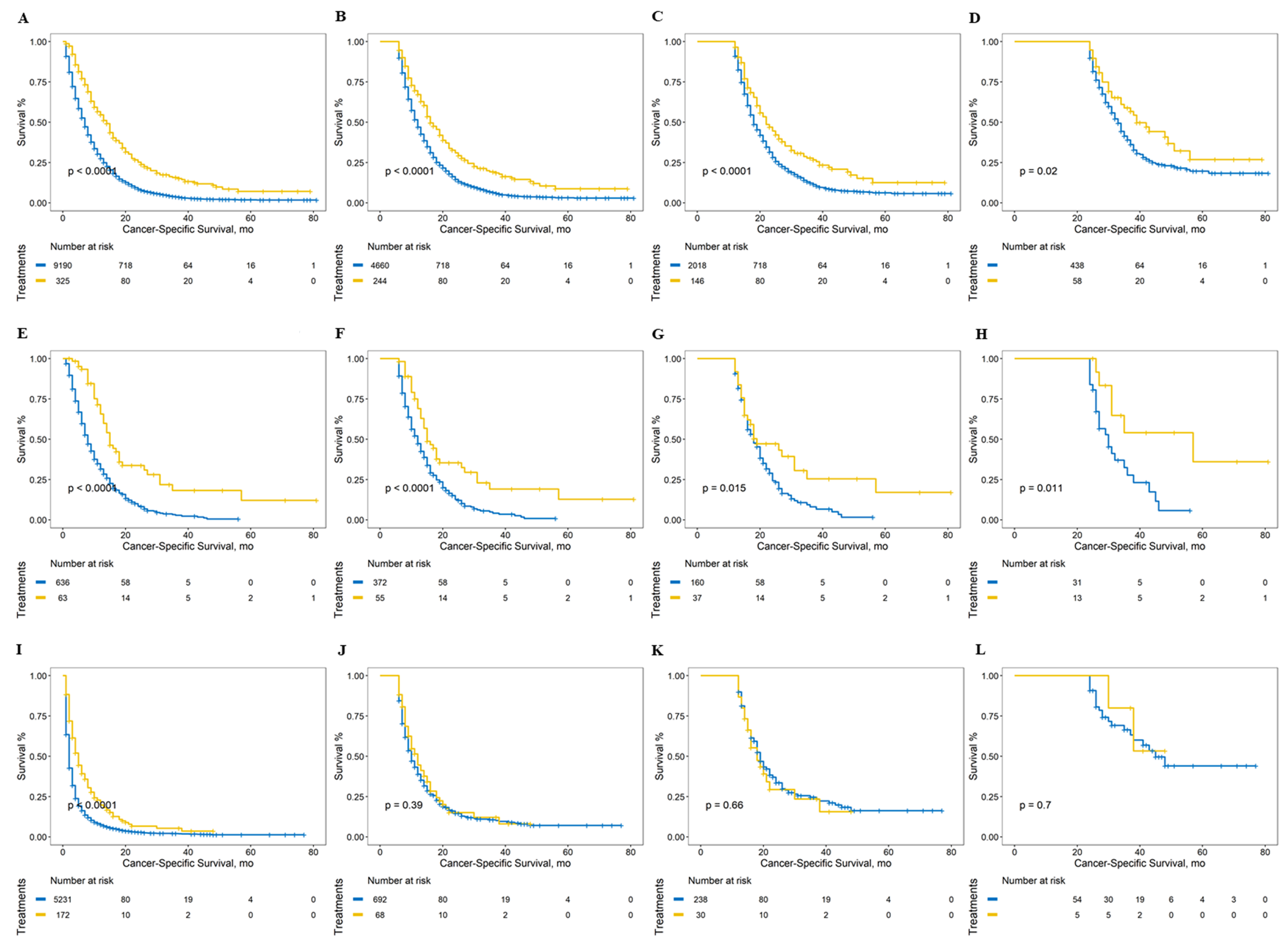

Supplement: Supplementary file 2 — Fig S2 [file CAM4-10-5948-s002.tif]
