# Supplementary material for: Worth it or not? Primary tumor resection for stage IV pancreatic cancer patients: A SEER‐based analysis of 15,836 cases
Source: Cancer Med. 2021 Jul 21;10(17):5948–63. doi: 10.1002/cam4.4147 (PMC8419755; doi:10.1002/cam4.4147)
Supplement: Supplementary file 3 — Table S1 [file CAM4-10-5948-s007.docx]

Supp. Table 1. Association between PTR-combined therapies(vs PTR-absence therapies) and overall survival analyzed via landmark analyses for patients confined to surviving for ≥0.6, ≥1, and ≥2 Years

|  |  | Group |  | Univariate Analysis | |  | Multivariate Analysis ^b^ | |  |
| --- | --- | --- | --- | --- | --- | --- | --- | --- | --- |
|  | No. of Patients | No. of Patients | No. of Patients |  |  |  |  |  |  |
| Landmark ^a^ | (no. of events) | (no. of events) | (no. of events) |  | 95% CI | *P* |  | 95% CI | *P* |
|  |  | Chemotherapy | PTR plus chemotherapy | HR With PTR plus chemo-therapy | | | aHR With PTR plus chemo-therapy | | |
| ≥6-month survivors | 4904 (4082) | 4660 (3897) | 244 (185) | 0.6 | (0.518-0.696) | <0.001 | 0.637 | (0.508-0.799) | <0.001 |
| ≥1-year survivors | 2164 (1721) | 2018 (1613) | 146 (108) | 0.617 | (0.507-0.750) | <0.001 | 0.639 | (0.467-0.875) | 0.005 |
| ≥2-year survivors | 496 (325) | 438 (291) | 58 (34) | 0.626 | (0.438-0.895) | 0.01 | 0.78 | (0.397-1.529) | 0.469 |
|  |  |  |  |  |  |  |  |  |  |
|  |  | No PTR | PTR | HR With PTR | | | aHR With PTR | | |
| ≥6-month survivors | 760 (663) | 692 (605) | 68 (58) | 0.833 | (0.636-1.091) | 0.185 | 1.005 | (0.627-1.612) | 0.983 |
| ≥1-year survivors | 268 (205) | 238 (183) | 30 (22) | 0.963 | (0.619-1.500) | 0.869 | 1.228 | (0.536-2.815) | 0.628 |
| ≥2-year survivors | 59 (30) | 54 (28) | 5 (2) | 0.598 | (0.142-2.515) | 0.483 | 4.14E+04 | (0-3.360E191) | 0.961 |
|  |  |  |  |  |  |  |  |  |  |
|  |  | Chemoradiotherapy | PTR plus chemoradiotherapy | HR With PTR plus chemoradiotherapy | | | aHR With PTR plus chemoradiotherapy | | |
| ≥6-month survivors | 427 (368) | 372 (328) | 55 (40) | 0.475 | (0.339-0.666) | <0.001 | 0.597 | (0.357-0.995) | 0.048 |
| ≥1-year survivors | 197 (164) | 160 (137) | 37 (27) | 0.563 | (0.366-0.864) | 0.009 | 0.655 | (0.334-1.285) | 0.219 |
| ≥2-year survivors | 44 (37) | 31 (29) | 13 (8) | 0.35 | (0.151-0.811) | 0.014 | 0.237 | (0.042-1.337) | 0.103 |
|  |  |  |  |  |  |  |  |  |  |

^a^ Landmarks analyses data are limited to patients surviving a minimum of ≥0.6, ≥1, and ≥2 years.

^b^ Multivariate aHRs are adjusted for the same factors shown either in Table2.

HR, hazard ratio. aHR, adjusted hazard ratio. CI, confidence interval. PTR, primary tumor resection.
