# Supplementary material for: Worth it or not? Primary tumor resection for stage IV pancreatic cancer patients: A SEER‐based analysis of 15,836 cases
Source: Cancer Med. 2021 Jul 21;10(17):5948–63. doi: 10.1002/cam4.4147 (PMC8419755; doi:10.1002/cam4.4147)
Supplement: Supplementary file 4 — Table S2 [file CAM4-10-5948-s004.docx]

Supp. Table 2. Association between PTR-combined therapies(vs PTR-absence therapies) and cancer-specific survival analyzed via landmark analyses for patients confined to surviving for ≥0.6, ≥1, and ≥2 Years

|  |  | Group |  | Univariate Analysis | |  | Multivariate Analysis ^b^ | |  |
| --- | --- | --- | --- | --- | --- | --- | --- | --- | --- |
|  | No. of Patients | No. of Patients | No. of Patients |  |  |  |  |  |  |
| Landmark ^a^ | (no. of events) | (no. of events) | (no. of events) |  | 95% CI | *P* |  | 95% CI | *P* |
|  |  | Chemotherapy | PTR plus chemotherapy | HR With PTR plus chemo-therapy | | | aHR With PTR plus chemo-therapy | | |
| ≥6-month survivors | 4904 (3774) | 4660 (3600) | 244 (174) | 0.613 | (0.526-0.714) | <0.001 | 0.635 | (0.502-0.802) | <0.001 |
| ≥1-year survivors | 2164 (1588) | 2018 (1483) | 146 (105) | 0.655 | (0.537-0.799) | <0.001 | 0.646 | (0.467-0.892) | 0.008 |
| ≥2-year survivors | 496 (295) | 438 (263) | 58 (32) | 0.657 | (0.454-0.949) | 0.025 | 0.737 | (0.366-1.483) | 0.392 |
|  |  |  |  |  |  |  |  |  |  |
|  |  | No PTR | PTR | HR With PTR | | | aHR With PTR | | |
| ≥6-month survivors | 760 (571) | 692 (518) | 68 (53) | 0.889 | (0.690-1.180) | 0.416 | 1.007 | (0.614-1.651) | 0.979 |
| ≥1-year survivors | 268 (173) | 238 (152) | 30 (21) | 1.105 | (0.700-1.744) | 0.669 | 1.191 | (0.496-2.863) | 0.695 |
| ≥2-year survivors | 59 (24) | 54 (22) | 5 (2) | 0.756 | (0.177-3.226) | 0.705 | 9686.92 | (0-7.770E137) | 0.953 |
|  |  |  |  |  |  |  |  |  |  |
|  |  | Chemoradiotherapy | PTR plus chemoradiotherapy | HR With PTR plus chemoradiotherapy | | | aHR With PTR plus chemoradiotherapy | | |
| ≥6-month survivors | 427 (330) | 372 (292) | 55 (38) | 0.516 | (0.365-0.730) | <0.001 | 0.695 | (0.408-1.183) | 0.18 |
| ≥1-year survivors | 197 (150) | 150 (125) | 37 (25) | 0.593 | (0.380-0.924) | 0.021 | 0.793 | (0.393-1.601) | 0.517 |
| ≥2-year survivors | 44 (30) | 31 (24) | 13 (6) | 0.314 | (0.118-0.831) | 0.02 | 0.373 | (0.048-2.879) | 0.344 |
|  |  |  |  |  |  |  |  |  |  |

^a^ Landmarks analyses data are limited to patients surviving a minimum of ≥0.6, ≥1, and ≥2 years.

^b^ Multivariate aHRs are adjusted for the same factors shown either in Table2.

HR, hazard ratio. aHR, adjusted hazard ration. CI, confidence interval. PTR, primary tumor resection.
