# Supplementary material for: Worth it or not? Primary tumor resection for stage IV pancreatic cancer patients: A SEER‐based analysis of 15,836 cases
Source: Cancer Med. 2021 Jul 21;10(17):5948–63. doi: 10.1002/cam4.4147 (PMC8419755; doi:10.1002/cam4.4147)
Supplement: Supplementary file 5 — Table S3 [file CAM4-10-5948-s006.docx]

Supp. Table 3. Baseline characteristics of IV PC patients receiving different surgery treatment modalities.

|  | Primary+distant/regional sites | Distant/regional site only | No surgery | Primary site only | p |
| --- | --- | --- | --- | --- | --- |
| n | 202 | 761 | 14513 | 360 |  |
| Age | 66.00 (59.00-72.75) | 67.00 (59.00-75.00) | 67.00 (59.00-75.00) | 66.00 (59.00-73.00) | 0.022 |
| Male (%) | 101 (50.0) | 356 (46.8) | 7772 (53.6) | 188 (52.2) | 0.003 |
| Race (%) |  |  |  |  | 0.113 |
| White | 161 (79.7) | 612 (80.4) | 11479 (79.1) | 297 (82.5) | |
| Black | 23 (11.4) | 104 (13.7) | 1938 (13.4) | 32 (8.9) |  |
| Other | 18 (8.9) | 45 (5.9) | 1096 (7.6) | 31 (8.6) |  |
| Grade (%) |  |  |  |  | <0.001 |
| 1 | 14 (6.9) | 14 (1.8) | 230 (1.6) | 22 (6.1) |  |
| 2 | 81 (40.1) | 73 (9.6) | 1316 (9.1) | 142 (39.4) | |
| 3 | 73 (36.1) | 71 (9.3) | 1560 (10.7) | 138 (38.3) | |
| 4 | 4 (2.0) | 3 (0.4) | 58 (0.4) | 0 (0.0) |  |
| Unknown | 30 (14.9) | 600 (78.8) | 11349 (78.2) | 58 (16.1) | |
| T (%) |  |  |  |  | <0.001 |
| 1 | 14 (6.9) | 49 (6.4) | 662 (4.6) | 25 (6.9) |  |
| 2 | 68 (33.7) | 246 (32.3) | 4465 (30.8) | 172 (47.8) | |
| 3 | 81 (40.1) | 150 (19.7) | 4358 (30.0) | 112 (31.1) | |
| 4 | 28 (13.9) | 128 (16.8) | 2236 (15.4) | 31 (8.6) |  |
| Unknown | 11 (5.4) | 188 (24.7) | 2792 (19.2) | 20 (5.6) |  |
| N (%) |  |  |  |  | <0.001 |
| 0 | 44 (21.8) | 42 (5.5) | 163 (1.1) | 73 (20.3) | |
| 1 | 74 (36.6) | 38 (5.0) | 119 (0.8) | 101 (28.1) | |
| 2 | 45 (22.3) | 5 (0.7) | 3 (0.0) | 115 (31.9) | |
| Unknown | 39 (19.3) | 676 (88.8) | 14228 (98.0) | 71 (19.7) | |
| Site (%) |  |  |  |  | <0.001 |
| bodytail | 95 (47.0) | 235 (30.9) | 5494 (37.9) | 89 (24.7) | |
| head | 81 (40.1) | 310 (40.7) | 4948 (34.1) | 235 (65.3) | |
| other | 12 (5.9) | 166 (21.8) | 2681 (18.5) | 20 (5.6) |  |
| overlap | 14 (6.9) | 50 (6.6) | 1390 (9.6) | 16 (4.4) |  |
| Msite bone (%) | 5 (2.5) | 45 (5.9) | 1072 (7.4) | 11 (3.1) | <0.001 |
| Msite brain (%) | 0 (0.0) | 10 (1.3) | 92 (0.6) | 0 (0.0) | 0.029 |
| Msite liver (%) | 92 (45.5) | 391 (51.4) | 11461 (79.0) | 207 (57.5) | <0.001 |
| Msite lung (%) | 18 (8.9) | 114 (15.0) | 2937 (20.2) | 38 (10.6) | <0.001 |
| number (mean (SD)) | 0.57 (0.57) | 0.74 (0.65) | 1.07 (0.61) | 0.71 (0.53) | <0.001 |
| Insurance (%) |  |  |  |  | 0.57 |
| Insured | 192 (95.0) | 733 (96.3) | 13925 (95.9) | 351 (97.5) | |
| Uninsured | 7 (3.5) | 22 (2.9) | 386 (2.7) | 6 (1.7) |  |
| unknown | 3 (1.5) | 6 (0.8) | 202 (1.4) | 3 (0.8) |  |
| Marital (%) | 131 (64.9) | 411 (54.0) | 8274 (57.0) | 240 (66.7) | <0.001 |
| Chemotherapy (%) | 134 (66.3) | 475 (62.4) | 9351 (64.4) | 254 (70.6) | 0.057 |
| Radiotherapy (%) | 26 (12.9) | 68 (8.9) | 785 (5.4) | 39 (10.8) | <0.001 |
