# Supplementary material for: Worth it or not? Primary tumor resection for stage IV pancreatic cancer patients: A SEER‐based analysis of 15,836 cases
Source: Cancer Med. 2021 Jul 21;10(17):5948–63. doi: 10.1002/cam4.4147 (PMC8419755; doi:10.1002/cam4.4147)
Supplement: Supplementary file 6 — Table S4 [file CAM4-10-5948-s003.docx]

Supp. Table 4. The pairwise comparisons of different organ involvement modalities based on log-rank test.

| **Pairwise Comparisons** | | | | | | | | | | | | | | | | | | | | |
| --- | --- | --- | --- | --- | --- | --- | --- | --- | --- | --- | --- | --- | --- | --- | --- | --- | --- | --- | --- | --- |
| Organ involvement code |  | **CSS** | | | | | | | | | | | | | | | | |  | |
|  |  | B | BC | BCH | BCHP | BCP | BH | BHP | BP | C | CH | CHP | CP | H | HP | Other | P |  | |  |
| OS | B |  | 0.084 | 0.010 | 0.051 | 0.329 | 0.000 | 0.000 | 0.697 | 0.616 | 0.003 | 0.047 | 0.694 | 0.056 | 0.000 | 0.115 | 0.130 |  | |  |
|  | BC | 0.223 |  | 0.526 | 0.951 | 0.048 | 0.469 | 0.619 | 0.123 | 0.067 | 0.779 | 0.959 | 0.389 | 0.163 | 0.403 | 0.026 | 0.023 |  | |  |
|  | BCH | 0.005 | 0.305 |  | 0.181 | 0.008 | 0.080 | 0.135 | 0.005 | 0.008 | 0.277 | 0.396 | 0.258 | 0.021 | 0.087 | 0.001 | 0.001 |  | |  |
|  | BCHP | 0.019 | 0.651 | 0.167 |  | 0.046 | 0.453 | 0.701 | 0.042 | 0.027 | 0.665 | 0.611 | 0.543 | 0.117 | 0.441 | 0.006 | 0.005 |  | |  |
|  | BCP | 0.438 | 0.659 | 0.134 | 0.416 |  | 0.109 | 0.041 | 0.414 | 0.373 | 0.037 | 0.049 | 0.221 | 0.229 | 0.102 | 0.505 | 0.523 |  | |  |
|  | BH | 0.001 | 0.668 | 0.032 | 0.174 | 0.921 |  | 0.139 | 0.006 | 0.062 | 0.172 | 0.356 | 0.499 | 0.000 | 0.858 | 0.000 | 0.000 |  | |  |
|  | BHP | 0.000 | 0.939 | 0.120 | 0.610 | 0.505 | 0.003 |  | 0.000 | 0.022 | 0.390 | 0.549 | 0.350 | 0.000 | 0.096 | 0.000 | 0.000 |  | |  |
|  | BP | 0.893 | 0.255 | 0.002 | 0.012 | 0.298 | 0.013 | 0.000 |  | 0.422 | 0.004 | 0.032 | 0.804 | 0.282 | 0.001 | 0.102 | 0.128 |  | |  |
|  | C | 0.496 | 0.084 | 0.001 | 0.003 | 0.379 | 0.063 | 0.008 | 0.329 |  | 0.007 | 0.015 | 0.476 | 0.284 | 0.072 | 0.920 | 0.970 |  | |  |
|  | CH | 0.000 | 0.404 | 0.230 | 0.637 | 0.348 | 0.016 | 0.222 | 0.000 | 0.000 |  | 0.823 | 0.271 | 0.015 | 0.178 | 0.000 | 0.000 |  | |  |
|  | CHP | 0.003 | 0.484 | 0.502 | 0.410 | 0.326 | 0.030 | 0.186 | 0.001 | 0.001 | 0.538 |  | 0.538 | 0.103 | 0.372 | 0.005 | 0.005 |  | |  |
|  | CP | 0.946 | 0.541 | 0.173 | 0.314 | 0.831 | 0.402 | 0.169 | 0.918 | 0.514 | 0.113 | 0.213 |  | 0.890 | 0.409 | 0.515 | 0.540 |  | |  |
|  | H | 0.564 | 0.258 | 0.005 | 0.018 | 0.441 | 0.000 | 0.000 | 0.795 | 0.372 | 0.000 | 0.002 | 0.906 |  | 0.000 | 0.000 | 0.000 |  | |  |
|  | HP | 0.001 | 0.569 | 0.034 | 0.141 | 0.865 | 0.729 | 0.000 | 0.009 | 0.097 | 0.013 | 0.030 | 0.415 | 0.000 |  | 0.000 | 0.000 |  | |  |
|  | Other | 0.003 | 0.061 | 0.000 | 0.000 | 0.129 | 0.000 | 0.000 | 0.014 | 0.925 | 0.000 | 0.000 | 0.526 | 0.000 | 0.000 |  | 0.969 |  | |  |
|  | P | 0.008 | 0.056 | 0.000 | 0.000 | 0.129 | 0.000 | 0.000 | 0.024 | 0.915 | 0.000 | 0.000 | 0.544 | 0.000 | 0.000 | 0.835 |  |  | |  |

Organ involvement code: P-lung, C-brain, H-liver, B-bone. The combination of the letters referred to multi-organ involvements. Other referred to those IV stage patients with metastatic organs other than 4 organs mentioned above. CSS outcome comparisons were marked as red background, while OS outcome comparisons were marked as blue background.
