# Supplementary material for: Worth it or not? Primary tumor resection for stage IV pancreatic cancer patients: A SEER‐based analysis of 15,836 cases
Source: Cancer Med. 2021 Jul 21;10(17):5948–63. doi: 10.1002/cam4.4147 (PMC8419755; doi:10.1002/cam4.4147)
Supplement: Supplementary file 7 — Table S5 [file CAM4-10-5948-s005.docx]

Supp. Table 5. Multivariate Cox regression analyses of different treatments stratified with different metastatic organ modalities considered for cancer-specific survival.

|  |  | PTR | | | Distant/reginal site resection | | | Radiotherapy | | | Chemotherapy | | |
| --- | --- | --- | --- | --- | --- | --- | --- | --- | --- | --- | --- | --- | --- |
|  | Number | aHR | 95%CI | P | aHR | 95%CI | P | aHR | 95%CI | Sig | aHR | 95%CI | P |
| B | 229 | 0.301 | 0.137-0.664 | **0.003** | 0.98 | 0.557-1.724 | 0.945 | 1.181 | 0.858-1.627 | 0.307 | 0.338 | 0.241-0.475 | **<0.001** |
| BC | 7 |  |  |  | 16.102 | 0.003-79865.239 | 0.522 | 2.007 | 0.178-22.642 | 0.573 | 0.003 | 0-370.28 | 0.333 |
| BCH | 6 |  |  |  | 1.003 | 0.089-11.344 | 0.998 | 33.639 | 0.01-116703.323 | 0.398 | 0.043 | 0-584.078 | 0.517 |
| BCHP | 14 |  |  |  | 8.65 | 0.512-146.198 | 0.135 | 0.16 | 0.028-0.901 | 0.038 | 0.216 | 0.04-1.175 | 0.076 |
| BCP | 8 |  |  |  | 2.823 | 0-1.479E+39 | 0.982 | 15.506 | 0-2.073E+40 | 0.952 | 0 | 0-4.426E+23 | 0.801 |
| BH | 444 | 0.59 | 0.08-4.333 | 0.604 | 0.905 | 0.438-1.871 | 0.788 | 0.842 | 0.651-1.088 | 0.189 | 0.365 | 0.289-0.461 | **<0.001** |
| BHP | 295 |  |  |  | 0.766 | 0.244-2.405 | 0.648 | 0.988 | 0.72-1.355 | 0.941 | 0.328 | 0.241-0.448 | **<0.001** |
| BP | 130 | 0.591 | 0.182-1.923 | 0.382 | 1.378 | 0.745-2.549 | 0.307 | 0.866 | 0.572-1.374 | 0.589 | 0.318 | 0.194-0.521 | **<0.001** |
| C | 15 |  |  |  | 0 | 0-3.511E+24 | 0.991 | 0.458 | 0.099-2.125 | 0.319 | 0.231 | 0.052-1.024 | 0.054 |
| CH | 26 |  |  |  | 0.5 | 0.054-4.599 | 0.54 | 1.104 | 0.388-3.142 | 0.853 | 0.605 | 0.221-1.659 | 0.329 |
| CHP | 14 |  |  |  | 0 | 0-4.127E10 | 0.984 | 0.113 | 0.008-1.542 | 0.102 | 0.989 | 0.135-7.253 | 0.992 |
| CP | 12 |  |  |  | 0 | 0-9.333E17 | 0.991 | 0.186 | 0.034-1.03 | 0.054 | 0.494 | 0.08-3.045 | 0.447 |
| H | 9657 | 0.561 | 0.488-0.646 | **<0.001** | 0.781 | 0.698-0.874 | **<0.001** | 0.859 | 0.758-0.974 | 0.018 | 0.412 | 0.393-0.433 | **<0.001** |
| HP | 1695 | 0.676 | 0.396-1.154 | 0.151 | 1.012 | 0.72-1.424 | 0.943 | 0.808 | 0.572-1.142 | 0.227 | 0.425 | 0.379-0.477 | **<0.001** |
| Other | 2345 | 0.635 | 0.536-0.752 | **<0.001** | 0.912 | 0.798-1.042 | 0.175 | 0.827 | 0.694-0.985 | 0.033 | 0.402 | 0.364-0.444 | **<0.001** |
| P | 939 | 0.729 | 0.5-1.063 | 0.101 | 0.883 | 0.656-1.187 | 0.409 | 0.972 | 0.684-1.382 | 0.875 | 0.477 | 0.407-0.559 | **<0.001** |
| aHR, adjusted hazard ratio. CI, confidence interval. P, p value.  Organ involvement code: P-lung, C-brain, H-liver, B-bone. The combination of the letters referred to multi-organ involvements. Other referred to those IV stage patients with metastatic organs other than 4 organs mentioned above.  Bold P value indicated those less than 0.01. | | | | | | | | | | | | | |
